# Rapid and reversible impairment of episodic memory by a high-fat diet in mice

**DOI:** 10.1038/s41598-018-30265-4

**Published:** 2018-08-10

**Authors:** Fiona H. McLean, Christine Grant, Amanda C. Morris, Graham W. Horgan, Alex J. Polanski, Kevin Allan, Fiona M. Campbell, Rosamund F. Langston, Lynda M. Williams

**Affiliations:** 1 Division of Neuroscience, University of Dundee, Ninewells Hospital and Medical School, Dundee, DD1 9SY UK; 2 Rowett Institute, University of Aberdeen, Foresterhill, Aberdeen, AB25 2ZD UK; 3 Biomathematics and Statistics Scotland, Aberdeen, AB25 2ZD UK; 4 School of Psychology, University of Aberdeen, Kings College, Old Aberdeen, AB24 3FX UK

## Abstract

Alzheimer’s disease is a leading cause of morbidity and mortality with no cure and only limited treatment available. Obesity and type 2 diabetes are positively associated with the development of premature cognitive decline and Alzheimer’s disease, linking diet with these conditions. Here we demonstrate that in mice episodic memory, together with spatial and contextual associative memory, is compromised after only one day of high-fat diet. However, object memory remains intact. This shows not only a more rapid effect than previously reported but also that more complex memories are at higher risk of being compromised by a high-fat diet. In addition, we show that these memory deficits are rapidly reversed by switching mice from a high-fat diet back to a low-fat diet. These findings have important implications for the contribution of nutrition to the development of cognitive decline and Alzheimer’s disease.

## Introduction

Alzheimer’s disease is one of the greatest global health challenges and the development of a successful cure has been hindered due to its complex aetiology. Nonetheless, being obese or having type 2 diabetes are known to be major risk factors for premature cognitive decline and the development of Alzheimer’s disease^1,2^. The Western diet, high in fat and sugar, is the lead driver in the development of obesity and type 2 diabetes and hence is inexorably linked to premature cognitive decline and Alzheimer’s disease^3^.

Previous research in rodents has shown that a high–fat diet (HFD) induces cognitive deficits after weeks or months of diet and has largely focused on spatial memory, tested using different forms of mazes^4^. In contrast, in this study we used multiple types of translational associative memory tasks prior to, during and after a HFD challenge to study the very early effects of HFD and the reversibility of these effects. The tasks used address object memory, spatial memory, contextual memory and episodic memory. Episodic memory is important in humans as it is one of the first types of memory to be compromised in Alzheimer’s disease^5^ and deficits in this memory type have been linked to a higher body mass index in young adults^6^. This is the first study to define the acute impact of a HFD on cognition using sensitive translational memory tasks^7,8^ in a well–defined rodent model of diet-induced obesity and glucose intolerance^9^.

**Figure 1:**
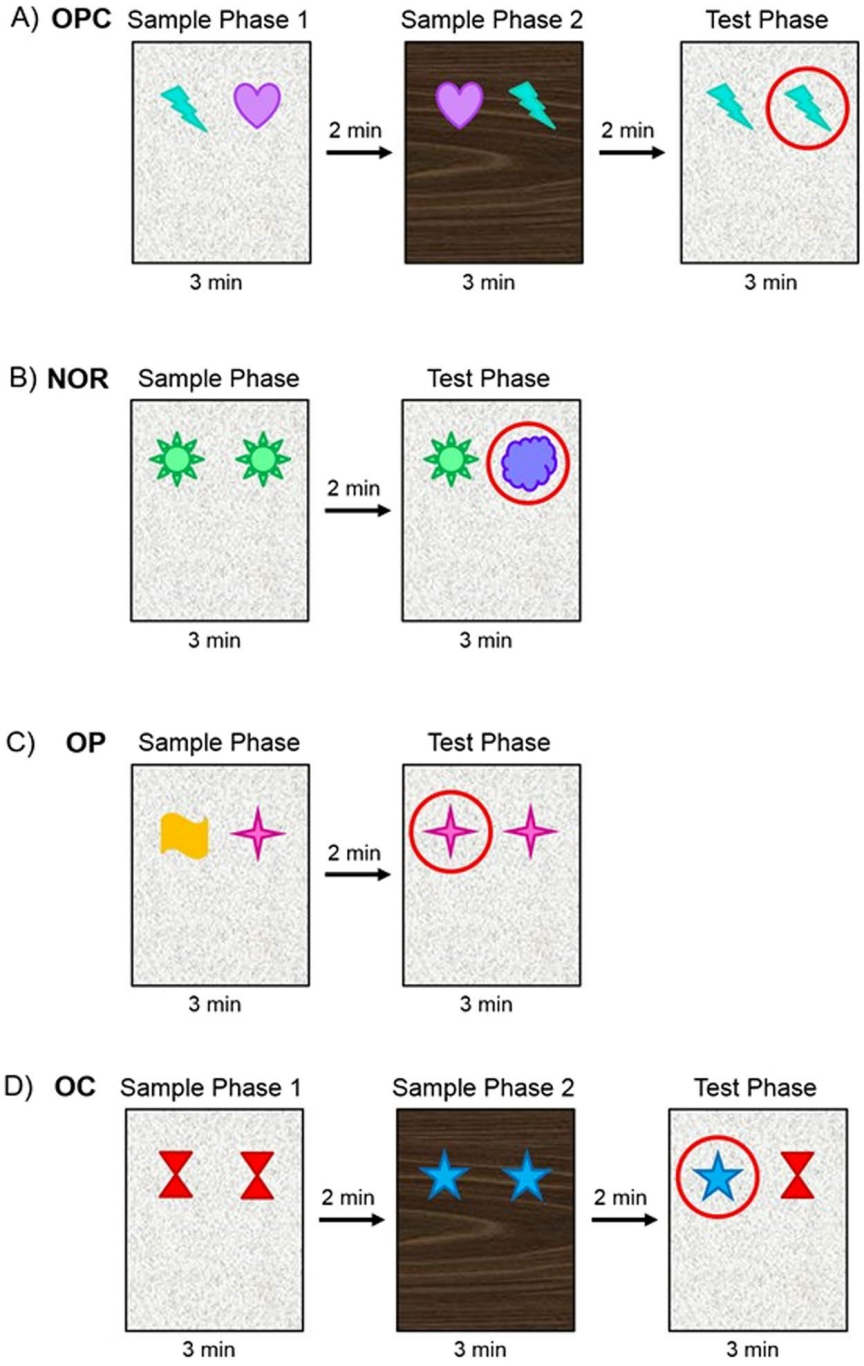
Schematic of the behavioural set-up used for different memory tasks. All tasks had phase lengths of 3 minutes with 2 minute intervals between phases. Objects which are novel or in a novel location/context for each task are circled. The four tasks are: **(A)** Object-Place-Context (OPC) task tests episodic memory. **(B)** Novel Object Recognition (NOR) task tests object memory. **(C)** Object-Place (OP) task tests spatial memory. **(D)** Object-Context (OC) task tests contextual memory.

Episodic memory encompasses unique “what, where, when/which” experiences that are spontaneously remembered^8,10^. To test episodic memory in mice we used the object-place-context task (Fig. 1A) which utilises the spontaneous exploration behaviour of rodents to investigate novel objects or environmental features^7^. This task requires mice to remember an object, where the object was seen and in which context from a single encoding event. Mice were also tested on spontaneous exploration tasks which test components of episodic memory. A novel object recognition (object memory) task tests the ‘what’ component (Fig. 1B), an object-place recognition (spatial memory) task tests the ‘where’ component (Fig. 1C), and an object-context recognition (contextual memory) task tests the ‘which’ component^7,11^ (Fig. 1D). Many brain areas have been found to have roles in episodic memory including the prefrontal cortex, cerebellum and the hippocampus and surrounding cortical areas^12,13^. In rodents, the hippocampus is key in the formation of episodic memory^14,15^, with rats bearing hippocampal lesions displaying deficits specifically in the object-place-context task^7,11^. Other component tasks do not require the hippocampus, with the novel object recognition task dependent on the perirhinal cortex^7,16,17^ and the spatial and contextual tasks dependent on the entorhinal and postrhinal cortices^18,19^. These areas of the brain input information to the hippocampus and are, thus, presumably also required for episodic memory formation^20,21^. Here we show that a HFD causes deficits in episodic, spatial and contextual memory within days after the start of the diet whilst novel object recognition remains intact. Additionally, we show that memory deficits can be reversed by a low-fat diet (LFD).

## Results

50% of the behavioural video data were blind scored by an independent observer. Blind scored data correlated highly with the original live scored data as follows (all n = 288): NOR (r = 0.82), OP (r = 0.80), OC (r = 0.74) and OPC (r = 0.79), all p < 0.001, confirming the validity of the results. Total object exploration data from the behavioural tasks is presented in Supplementary Information, Fig. S1.

### High-fat diet causes rapid deficits in episodic memory

**Figure 2:**
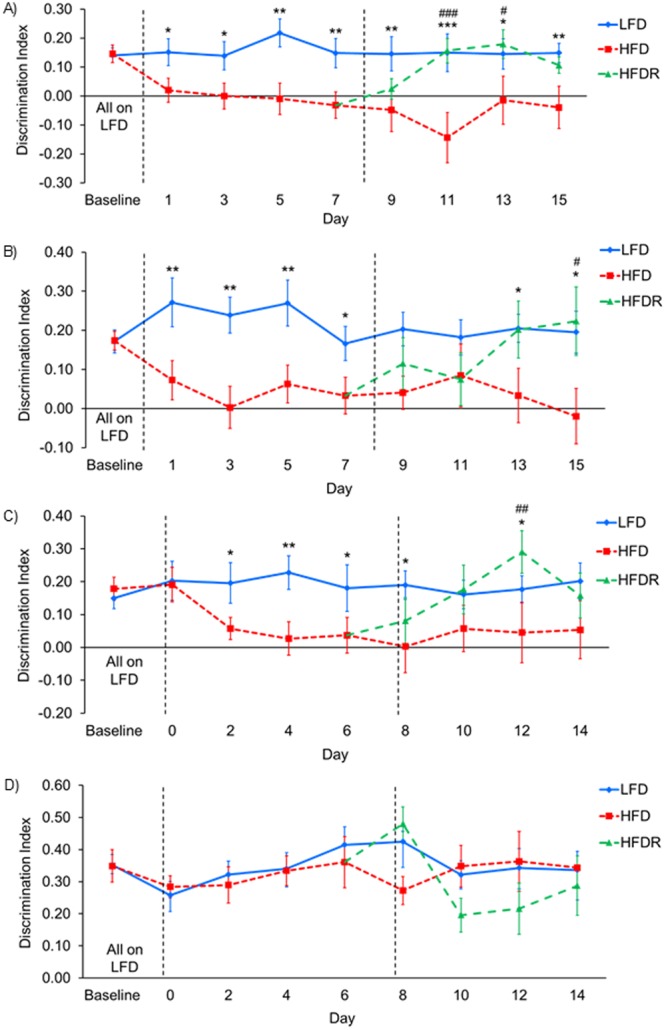
Behavioural memory task results. **(A)** Object-Place-Context (OPC) task. Linear mixed model for repeated measures (LMMRM) showed an effect of diet (F_(2, 125.263)_ = 14.821, p < 0.001), time (F_(7,196.519)_ = 2.451, p = 0.02) and no interaction (F_(10,201.976)_ = 0.767, p = 0.66). HFD mice performed significantly worse than LFD mice on all days tested. HFDR mice performed significantly better than HFD mice on days 11 and 13. **(B)** Object-Place (OP) task. LMMRM showed an effect of diet (F_(2,161.124)_ = 10.315, p < 0.001), not time (F_(7,275.612)_ = 0.668, p = 0.699) and no interaction (F_(10, 270.500)_ = 1.319, p = 0.220). HFD mice performed significantly worse than LFD mice on all days tested except days 9 and 11. HFDR mice performed significantly better than HFD mice on day 15. **(C)** Object-Context (OC) task. LMMRM showed an effect of diet (F_(2, 135.472)_ = 9.219, p < 0.001), not time (F_(7, 209.121)_ = 0.312, p = 0.948) and no interaction (F_(10, 229.941)_ = 0.427, p = 0.932). HFD mice performed significantly worse than LFD mice on all days tested except days 10 and 14. HFDR mice performed significantly better than HFD mice on day 12. **(D)** Novel Object Recognition (NOR) task. Linear mixed model for repeated measures showed no effect of diet (F_(2,177.697)_ = 1.379, p = 0.255), an effect of time (F_(7,257.597)_ = 2.253, p = 0.031) and no interaction (F_(10,252.918)_ = 1.650, p = 0.093). All data are mean ± SEM. LFD *vs*. HFD *p < 0.05, **p < 0.01, ***p < 0.001; HFD *vs*. HFDR ^#^p < 0.05, ^##^p < 0.01, ^###^p < 0.001. LFD group n = 24, HFD group n = 24 until day 8 where group was split into HFD n = 12 and HFDR group n = 12. Dashed lines indicate the day of diet change but are slightly offset to avoid overlying data points. Low-fat diet (LFD), high-fat diet (HFD), high-fat diet recovery (HFDR) and linear mixed model for repeated measures (LMMRM).

Following the switch to a HFD there was a rapid drop in the performance in the episodic memory task. Performance deficits were seen after only one day of HFD and these deficits were sustained on every subsequent day the mice were tested (Fig. 2A), showing that a HFD rapidly compromises hippocampal dependent episodic memory.

### High-fat diet causes concurrent deficits in spatial and contextual memory whilst object memory is maintained

In order to investigate if a HFD causes deficits solely in episodic memory or whether the components of episodic memory are also inhibited, object memory, spatial memory and contextual memory tasks were tested alongside the episodic memory task. The decreased ability of HFD mice to perform the episodic memory task occurred concurrently with deficits in the spatial memory task (Fig. 2B) and the contextual memory task (Fig. 2C). All groups of mice performed the object memory task equally well (Fig. 2D).

### Switching from a high-fat diet back to a low-fat diet reverses cognitive deficits

After establishing that a HFD induced rapid deficits in episodic, spatial and contextual memory, we investigated whether these deficits could be reversed. After 8 days on a HFD half the mice were switched back to a LFD for an additional 8 days; termed high-fat diet recovery (HFDR). In the episodic memory task the impairment in the HFDR group was reversed while HFD mice maintained the deficit (Fig. 2A). In the spatial and contextual memory tasks, the impairment in the HFDR group was also reversed while HFD mice maintained the deficits (Fig. 2B,C). The HFDR and HFD groups maintained the ability to perform the object memory task throughout (Fig. 2D).

### Memory deficits occur alongside increases in body weight, fat mass and the development of glucose intolerance

**Figure 3:**
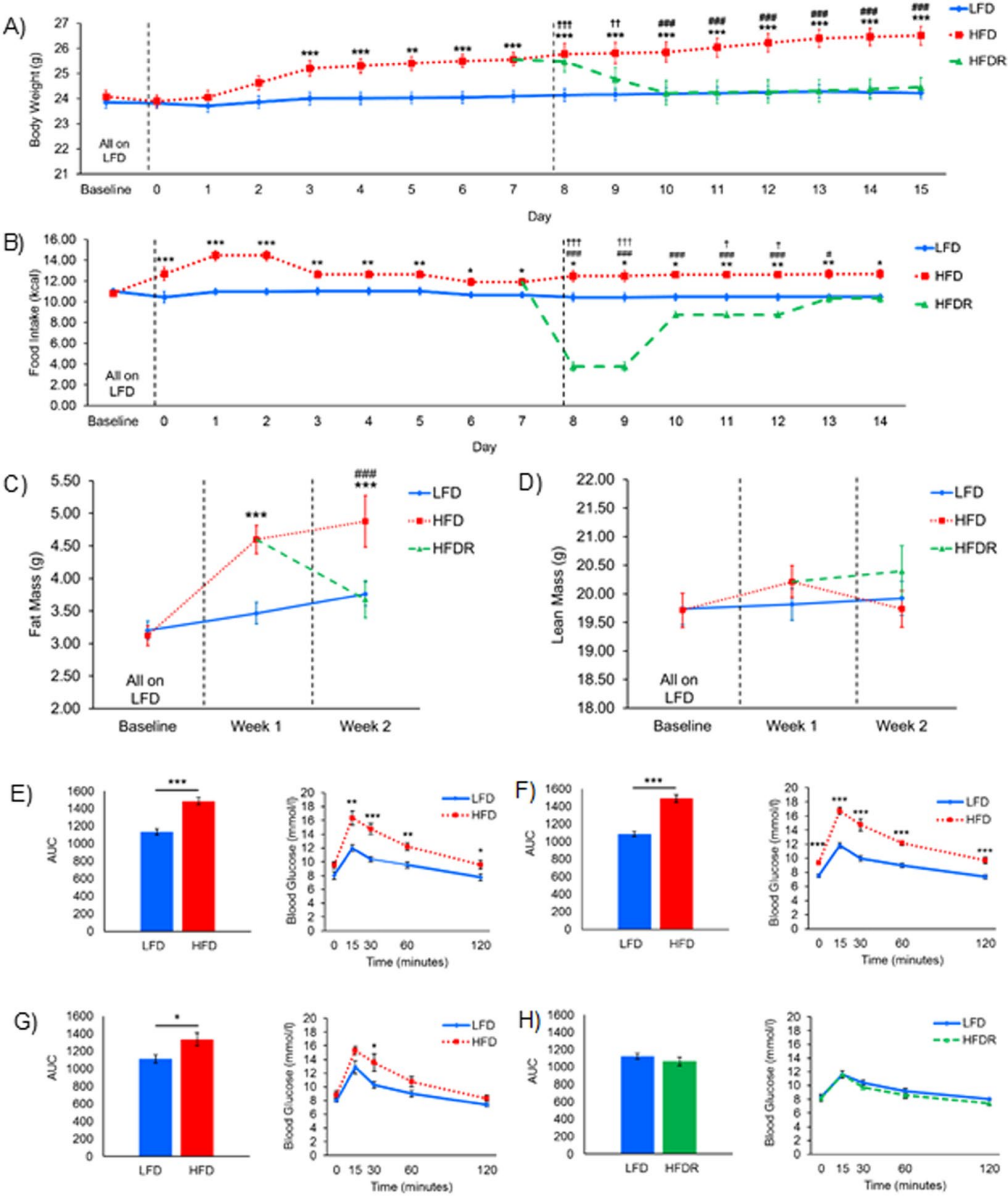
Body weight, composition and food intake. **(A)** Body weight. LMMRM showed an effect of diet (F_(2,160.295)_ = 42.785, p < 0.001), time (F_(16,669.882)_ = 21.131, p < 0.001) and an interaction (F_(23,675.798)_ = 10.772, p < 0.001). HFD mice were heavier than LFD mice from day 3 onwards. HFDR mice decreased body weight following the switch to LFD, and from 2 days after were comparable with LFD mice. **(B)** Food intake. LMMRM showed an effect of diet (F_(2,156.363)_ = 72.014, p < 0.001), time (F_(15,423.506)_ = 6.676, p < 0.001) and an interaction (F_(21,509.623)_ = 5.582, p < 0.001). HFD mice consumed more calories than LFD mice throughout. HFDR mice showed a reduction in caloric intake compared to HFD and LFD mice, which recovered by day 13 to LFD levels. **(A**,**B)** Day 1–7: LFD n = 24, HFD n = 24. Day 8–15: LFD n = 24, HFD n = 12, HFDR n = 12. **(C)** Fat mass. LMMRM showed an effect of diet (F_(2,109.449)_ = 23.860, p < 0.001), time (F_(2,88.941)_ = 62.554, p < 0.001) and an interaction (F_(2,88.941)_ = 26.218, p < 0.001). HFD mice had higher fat mass than LFD mice in weeks 1 and 2. HFDR mice had lower fat mass than HFD mice and equivalent fat mass to LFD mice in week 2. **(D)** Lean mass. LMMRM showed no effect of diet (F_(2,105.154)_ = 0.664, p = 0.517), an effect of time (F_(2,87.590)_ = 4.174, p = 0.019) and no interaction (F_(2,87.590)_ = 2.205, p = 0.116). **(C**,**D)** Baseline & week 1: LFD n = 23, HFD n = 23; Week 2: LFD n = 23, HFD n = 12, HFDR n = 11. **(E–H)** Intraperitoneal glucose tolerance tests (IPGTTs). One-way ANOVA on AUC showed a difference between LFD and HFD mice at (E) 3 days (F_(1,14)_ = 42.519, p < 0.001), (F) 1 week (F_(1,14)_ = 62.827, p < 0.001) and (G) 2 weeks (F_(1,14)_ = 5.894, p = 0.029) but not (H) between LFD and HFDR at 2 weeks (F_(1,14)_ = 0.998, p = 0.335). Blood glucose levels in LFD vs HFD mice at (E) 3 days, (F) 1 week and (G) 2 weeks and (H) LFD vs HFDR mice at 2 weeks. All groups n = 8. **(A–H)** Data are mean ± SEM. LFD vs HFD *p < 0.05, **p < 0.01, ***p < 0.001; LFD vs HFDR ^†^p < 0.05, ^††^p < 0.01, ^†††^p < 0.001; HFD vs HFDR ^#^p < 0.05, ^##^p < 0.01, ^###^p < 0.001. Dashed lines indicate diet switch. Diets: Low-fat (LFD), high-fat (HFD), high-fat recovery (HFDR), area under curve (AUC) and linear mixed model for repeated measures (LMMRM).

Memory deficits in HFD mice occurred concurrently with increased body weight and memory deficits were reversed alongside body weight loss in HFDR mice (Fig. 3A). Caloric intake was higher in HFD mice (Fig. 3B). Following the switch back to LFD, the HFDR mice showed a significant reduction in caloric intake compared to not only HFD mice but also compared to LFD mice. This drop in caloric intake recovered over the following days to LFD levels.

To confirm that body weight fluctuations were due to changes in fat mass, body composition was measured by Echo MRI (Fig. 3C). Fat mass increased in HFD mice compared to LFD mice in weeks 1 and 2. Fat mass decreased in HFDR mice after the switch back to LFD. Lean mass was unchanged between the diet groups throughout (Fig. 3D).

In intraperitoneal glucose tolerance tests (IPGTTs) after 3 days, 1 week and 2 weeks of HFD (Fig. 3E–G), blood glucose levels were significantly higher in HFD than LFD mice. Following the switch back to LFD, HFDR mice showed blood glucose levels equivalent to that of LFD mice (Fig. 3H). Body weights and food intakes of mice used for IPGTT studies can be found in Supplementary Information (Fig. S2). Detailed statistical analysis of IPGTT data can also be found in Supplementary Information.

## Discussion

Our study shows that HFD-induced cognitive deficits in complex episodic and associative memories occur rapidly, clearly demonstrating that the process of diet-induced cognitive dysfunction in rodents starts much earlier than previously shown^3,4^. The rapid impairments in spatial, contextual and episodic memory tasks suggest HFD-induced cognitive dysfunction in the postrhinal and entorhinal cortices and hippocampus. However there was no change in the performance of the object memory task thought to be processed by the perirhinal cortex. Other studies using NOR tasks to test the effect of a HFD on object memory in C57Bl/6J mice have found diverse effects^4^. These differences may be due to different amounts of fat in the HFD (i.e. 40% or 60% energy from fat), different forms of the NOR tests used or different retention intervals between the NOR sample and test phases since longer retention intervals may require the hippocampus^22^.

In our study the loss of ability to perform certain tasks while others are unaffected implies brain region specific effects of a HFD. If these deficits also occur in humans, this would support the theory that a high-fat diet may contribute to early cognitive decline leading to the development of Alzheimer’s disease^6,23^. The loss of episodic memory is a defining feature of early onset Alzheimer’s disease and the memory tasks which show deficits in our study utilise the entorhinal cortex and hippocampus, two areas of the brain which are affected early in Alzheimer’s disease^24,25^. Interestingly, the 3xTgAD mouse model of Alzheimer’s disease also demonstrates deficits in the OPC task^26^. Additionally, SAMP8 mice, which exhibit some of the pathological signs of Alzheimer’s disease, have episodic memory deficits rescued by acarbose, a drug used to treat type-2 diabetes, supporting the link between Alzheimer’s disease and metabolism^27^. Furthermore, we have identified these HFD-induced memory deficits in mice considered “young adults” suggesting that diet can impact on memory at a relatively early age. Deficits in episodic memory in humans have been associated with a higher body mass index in young adults^6^.

It should be noted that obesity and type 2 diabetes are also risk factors for the development of vascular dementia as well as Alzheimer’s disease^28^. Distinguishing between these two diseases can be challenging since symptoms can be similar. When hyperglycemia occurs in the brain it causes neuroinflammation, increases in areas of white matter hyper-intensity associated with Aβ deposition as well as microvascular disease^2^. Patients suffering from vascular dementia with microvascular pathology have been shown to have hippocampal atrophy^29^. Our study may also be relevant to the cognitive deficits induced by a high-fat diet which may be a prelude to the development of vascular dementia.

The memory deficits identified in the present study are also rapidly reversed by the return to a LFD. This indicates that a balanced diet in which high-fat meals are interspersed with lower fat meals may equilibrate episodic memory function. Nonetheless, this also implies that an unremitting intake of a HFD can result in cognitive decline. While this is the first study investigating memory recovery from the acute impact of HFD, it has been reported calorie restriction in mice can improve cognition^30^. Furthermore, in humans, calorie restriction can improve cognition in the elderly^31^. The high-fat recovery data supports the fact that reducing fat consumption can be beneficial to cognitive function.

While the present study does not address mechanisms underlying the effect of HFD on memory it has been postulated previously that these may include increased adiposity and glucose intolerance. This is directly supported by the present data where glucose intolerance coincides with the development of episodic, spatial and contextual memory deficits and recovery of glucose tolerance occurs alongside recovery of memory. Also, studies have implicated both insulin and leptin insensitivity in the development of memory deficits and while this was not tested in the present study it is widely reported that both insulin and leptin insensitivity develop rapidly in mice on a HFD^9,32^. Indeed insulin resistance in the brain has been shown to occur rapidly in rodents after only 3 days of a HFD^33,34^. Insulin acts on pyramidal neurons in the hippocampus to enhance hippocampal long-term potentiation^35^ and the modulation of synaptic plasticity^36,37^. Insulin resistance has been demonstrated in the brain in Alzheimer’s disease^38^ and enhancing insulin signalling in the brain improves learning and memory^39,40^. Furthermore drugs used to treat type II diabetes, either via improving insulin sensitivity or increasing the release of insulin, impact positively on learning and memory^41,42^. Leptin has also been implicated in cognition with leptin receptors in the hippocampus involved in neuronal excitability and leptin-deficient rodents showing impaired spatial memory^43,44^. Leptin treated mouse models of Alzheimer’s disease show a reduction of β-amyloid and phosphorylated tau, as well as improvements in performance of learning and memory tasks^45^. The dramatic drop in food intake on days 8 and 9 by the HFDR group could be a result of switching to, what the mice may perceive as, a relatively less palatable diet or due to a rapid re-establishment of leptin sensitivity after HFD-induced leptin insensitivity^32^. This would coincide with the improvement in memory task performance. Thus, HFD-induced insensitivity to both insulin and leptin may impact on memory.

In conclusion our study demonstrates a rapid and reversible region specific influence of HFD on memory and provides the basis for mechanistic studies into how a HFD can so rapidly compromise complex episodic and related memory types while leaving object memory unimpaired. The rapid onset and subsequent reversal of HFD-induced memory deficits indicate the speed at which diet can influence cognition and supports the relationship between diet, obesity and type 2 diabetes. Our data also supports dietary advice that reducing saturated fat consumption is a viable approach to healthier brain aging.

### Contact for reagent and resource sharing

Further information and requests for reagents may be directed and will be fulfilled by the corresponding author Fiona H. McLean (f.mclean@dundee.ac.uk).

## Methods

### Animals

Male, 12 week old, C57Bl/6J mice (Harlan Laboratories UK) were used. All studies adhered to UK Home Office regulations according to the Animals (Scientific Procedures) Act, 1986, were in accordance with the European Directive on the Protection of Animals used for Scientific Purposes 2010/63/E, and followed ARRIVE guidelines. Experimental protocols were approved by the Rowett Institute’s Ethical Review Committee. Animals were singly housed on grid floors and maintained on a 12:12 hour light:dark cycle with access to food and water *ad libitum*. Environmental enrichment was provided. Mice were counterbalanced between groups based on body weight at the beginning of the experiment.

### Experimental diets and study designs

Low-fat diet (10% energy from fat) D12450B and high-fat diet (60% energy from fat) D12492 (Research Diets Inc. New Jersey, US) were used. Mice were fed a LFD for 8 days to acclimatise to a semi-purified diet and then either remained on the LFD for a further 2 weeks, were switched to a HFD for 2 weeks or were fed a HFD for 1 week then switched back to a LFD for a week (high-fat diet recovery (HFDR)). A total of n = 112 mice were used with 48 mice undergoing behavioural testing and 64 mice used for intraperitoneal glucose tolerance testing (IPGTT). At the end of the experiment mice were killed by exsanguination under terminal anaesthesia.

### Body weight, body composition and food intake measurements

Body weight and food intake were measured 3 times weekly and on the day of diet switches. Body composition was measured once a week using an Echo MRI 2013 Body Composition Analyser version 130118 with two mice being excluded as they showed signs of distress.

### Intraperitoneal glucose tolerance test (IPGTT)

Mice were fasted for 6 hours, then injected intraperitoneally with glucose (1.5 mg/g body weight). Glucose levels were measured in tail vein blood using an Accu-Chek Aviva glucose monitor at 0, 15, 30, 60 and 120 minutes. At the end of the experiment mice were killed as detailed above.

### Behavioural memory tasks

Testing was carried out in a rectangular arena 31 cm by 45 cm with 35 cm high walls. Two different contexts were used. Context 1 had a light brown wood-effect floor and dark brown wood-effect walls. Context 2 had a grey grid floor and white marble-effect walls. Dual Lock (3 M, Bracknell, UK) adhesive strips were secured at two positions on the floor 15 cm from the North wall, 9 cm from the edge of the arena and 13 cm apart. Objects, composed of non-porous substances (e.g. glass, plastic or metal) and a maximum of 15 cm high, 15 cm long and 10 cm wide, were attached to the Dual-Lock strips. Two constant visual cues were secured in the top left and right corners.

Mice were handled daily for 2 weeks and habituated to the arena for 1 week. Habituations rotated order of contexts followed by the introduction of identical and non-identical pairs of objects in the arena. All behavioural tasks were carried out in the light phase and were tested every second day: novel object recognition and object-context tasks were tested one day followed by object-place and object-place-context tasks the next. Each task consisted of either one or two sample phases followed by a test phase. Before and between phases mice were placed in a holding box. All phases lasted 3 minutes with two minute intervals between. Mice were placed in the arena from the South and facing the South wall. The arena and objects were cleaned using lemon scented anti-bacterial wipes between phases. To remove any bias of an object pair, position within the arena or a context, the novel object in the task, the position of the novel object and the context that the novel object appeared in was counterbalanced across mice. To remove any potential bias of an individual object from residual odour marking or local cues, animals were presented with an identical copy in each phase.

#### Object exploration

Exploration of an object was defined as actively engaging with an object (sniffing, close inspection, investigating and showing interest). Sitting on or near the object without engaging and compulsive chewing of an object was not classed as exploration. A minimum of 5 seconds exploring each object in the sample phases and a minimum of 10 seconds total exploration of objects in the test phase was required as a minimum. If these criteria were not met then data were excluded.

The time spent exploring was converted into a discrimination index (DI) calculated using the formula (time spent exploring novel object – time spent exploring familiar object) / (time spent exploring novel object + time spent exploring familiar object). ‘Novel’ refers either to the novel object (NOR), the object in a novel location (OP), the object in a novel context (OC) or the object in a novel position-context configuration (OPC) in the test phase. The familiar object is presented in the test phase. A value of 0 means equal exploration of both objects. A positive value corresponds to more exploration of the novel object whilst a negative value corresponds to preferential exploration of the familiar object. A DI greater than 0 indicates a memory of the familiar object. Use of this formula normalises data between animals.

For all tasks the total length of time spent exploring objects in the sample phase(s) and test phase was compared between diet groups (Supplementary Information Fig. S1) to ensure that any differences in task performance were not due to differences in the total length of time spent exploring.

#### Object-place-context (OPC) memory task

The OPC task^8^ consists of two sample phases followed by a test phase (Fig. 1A). The two objects presented in the test phase are identical, however, one, whilst familiar in its position and context, is novel in its position-context configuration. Increased exploration of this novel object indicates intact episodic memory.

#### Novel object recognition (NOR) memory task

The NOR task^46^ consists of a sample phase followed by a test phase (Fig. 1B) where a familiar object and a ‘novel’ object are presented. Greater exploration of the novel object indicates that mice remember the familiar object and that object memory is intact.

#### Object-place (OP) memory task

The OP task^11^ consists of a sample phase followed by a test phase (Fig. 1C) where one of the objects is in a new position and is thus seen as ‘novel’. Increased exploration of the object in a novel position indicatives functioning spatial memory.

#### Object-context (OC) memory task

The OC task^11^ consists of two sample phases followed by a test phase (Fig. 1D) where one object, whilst familiar in position, is novel in context. Increased exploration of this object signifies that contextual memory is intact.

All behavioural data was recorded using an overhead camera (Ganz FCH-64 Hi-Res 570 TVL B/W 1/3′′ CS Mount Camera, Fujinon Vari-Focal CCTV Lens) and tasks were scored using AnyMaze software (Stoelting Co., Illinois, USA). Exploration was scored live via the video output. 50% of video recordings were scored blind by an independent observer.

### Statistical analysis

Behavioural data was analysed using a linear mixed model for repeated measures (LMMRM). Fixed effects were diet and time. The baseline DI were used as a covariate for each mouse and an autoregressive order 1 model for the correlation between time points was included. Fisher’s LSD corrected pairwise simple effects were calculated when an interaction of main effects was found. Blind scored data were compared with original data using Pearson’s Correlation (r) coefficient.

Total exploration, body weight, composition and food intake data were analysed using LMMRM. Fixed effects were diet and time. An autoregressive order 1 model for the correlation between time points was included. Fisher’s LSD corrected pairwise simple effects were calculated when an interaction of main effects was found.

Blood glucose data were analysed using a general linear model for repeated measures with Fisher’s LSD corrected pairwise simple effects calculated between diet groups at each time point during the IPGTT. AUC was calculated and compared by one-way ANOVA.

Data are reported as mean ± SEM. The threshold for statistical significance is set at p < 0.05. All statistical analysis was carried out using SPSS (Statistical Package for the Social Sciences, USA)

### Data availability

The datasets generated during and/or analysed during the current study are available from the corresponding author on reasonable request.

## Electronic supplementary material

Supplementary Information
