# Supplementary material for: Rapid and reversible impairment of episodic memory by a high-fat diet in mice
Source: Sci Rep. 2018 Aug 10;8:11976. doi: 10.1038/s41598-018-30265-4 (PMC6086894; doi:10.1038/s41598-018-30265-4)
Supplement: Supplementary file 1 — Supplementary Information [file 41598_2018_30265_MOESM1_ESM.docx]

Rapid and reversible impairment of episodic memory by a high-fat diet in mice.

Fiona H. McLean^1,2^*, Christine Grant^2^, Amanda C. Morris^2^, Graham W. Horgan^3^, Alex J. Polanski^2^, Kevin Allan^4^, Fiona M. Campbell^2^, Rosamund F. Langston^1#^ and Lynda M. Williams^2#^.

^1^ Division of Neuroscience, University of Dundee, Ninewells Hospital and Medical School, Dundee, DD1 9SY. ^2^ Rowett Institute, University of Aberdeen, Foresterhill, Aberdeen, AB25 2ZD, UK. ^3^ Biomathematics and Statistics Scotland, Aberdeen, AB25 2ZD, UK. ^4^ School of Psychology, University of Aberdeen, Kings College, Old Aberdeen, AB24 3FX, UK. *corresponding author and lead contact, ^#^equal contribution.

**supplementary information**

**Behavioural Data**

***Total object exploration***

Total object exploration showed little or no differences between the diets. There was an effect of time in the NOR sample (F_(8,264.603)_=2.485, p<0.001) and test (F_(8,268.171)_=34.727, p<0.001) phases, no effect of diet and no interaction. The LFD group explored more than the HFD group on day 2 (p=0.008) and the HFDR group explored less than the HFD group on day 10 (p=0.046) (Figure S1B). There was an effect of time in the OP sample (F_(8,256.609)_=31.389, p<0.001) and test (F_(8,253.256)_=7.166, p<0.001) phases, no effect of diet and no interaction (Figure S1C and D). In the first OC sample phase there was an effect of time (F_(8,260.245)_=6.980, p<0.001), and diet (F_(2,165.100)_=4.512, p=0.012) and no interaction. The HFDR group explored less than the LFD group on day 12 (p=0.009) and the HFD group explored less than the LFD group on day 0 (p=0.007) and day 2 (p=0.014) (Figure S1E). In the second OC sample phase there was an effect of time (F_(8,255.737)_=7.888, p<0.001), no effect of diet (F_(2,163.760)_=2.499, p=0.085) and no interaction (Figure S1F). In the OC test phase there was an effect of time (F_(8,254.661)_=5.992, p<0.001), no effect of diet and no interaction (Figure S1G). In the first OPC sample phase there was an effect of time (F_(8,240.741)_=25.231, p<0.001), no effect of diet and no interaction (Figure S1H). In the second OPC sample phase there was an effect of time (F_(8,250.542)_=15.133, p<0.001), an effect of diet (F_(2,155.216)_=3.293, p=0.040) and no interaction. The HFD group explored less than the LFD group on day 5 (p=0.041) and the HFDR group explored less than the LFD group on day 11 (p=0.034) (Figure S1I). In the OPC test phase there was an effect of time (F_(8,250.145)_=3.972, p<0.001), no effect of diet and no interaction (Figure S1J). Exploration for all tasks showed fluctuation over time due to habituation to the tests and intrinsic preferences for objects pairs presented on certain days. Any differences between diet groups were not deemed to impact on task performance.

**
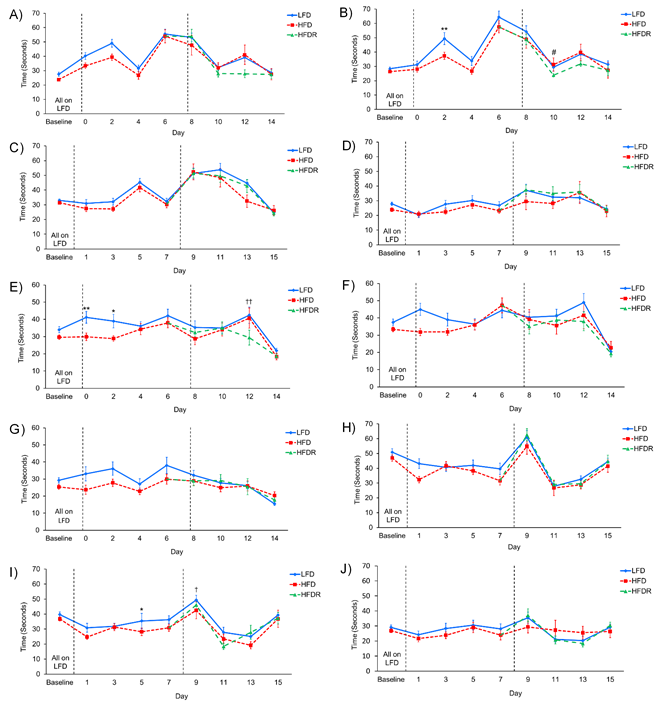
**

**Figure S1** - Total object exploration for all tasks: A) NOR sample phase. B) NOR test phase. C) OP sample phase. D) OP test phase. E) OC sample phase 1. F) OC sample phase 2. G) OC test phase. H) OPC sample phase 1. I) OPC sample phase 2. J) OPC test phase. All data are mean ± SEM. LFD *vs.* HFD * p<0.05, ** p<0.01, *** p<0.001; HFD *vs.* HFDR # p<0.05, ## p<0.01, ### p<0.001. LFD *vs.* HFDR † p<0.05, †† p<0.01, ††† p<0.001. LFD group n=24, HFD group n=24 until day 8 where group was split into HFD n=12 and HFDR group n=12. Vertical dashed lines indicate the day of diet change but are offset to avoid overlying data points. Low-fat diet (LFD), high-fat diet (HFD), high-fat diet recovery (HFDR), novel object recognition (NOR), object-place (OP), object-context (OC) and object-place-context (OPC).

**Intraperitoneal Glucose Tolerance Test (IPGTT)**

***Glucose Tolerance over Time on Diet***

After 3 days on HFD, general linear model for repeated measures showed an effect of diet (F_(1,14)_=45.032, p<0.001), time (F_(4,56)_=34.961, p<0.001) and an interaction (F_(4,56)_=2.913, p=0.029). After 1 week on HFD, general linear model for repeated measures showed an effect of diet (F_(1,14)_=73.005, p<0.001), time (F_(2.503,35.038)_=89.148, p<0.001) and an interaction (F_(2.503,35.038)_=7.171, p=0.001). After 2 weeks on HFD, general linear model for repeated measures showed an effect of diet (F_(1,14)_=6.167, p=0.026), time (F_(2.577,36.074)_, p<0.001) and no interaction (F_(2.577,36.074)_, p=0.103). In HFDR mice 1 week following the return to LFD, general linear model for repeated measures showed an effect of time (F_(4,56)_=64.209, p<0.001), no effect of diet (F_(1,14)_=0.626, p=0.442) and no interaction (F_(4,56)_=0.606, p=0.660).

***Mice Body Weights and Food Intakes***

Body weight and food intake data for mice used for IPGTT was similar to that of mice used for behavioural studies. Detailed analysis of IPGTT mice can be found in Figure S2A-H.


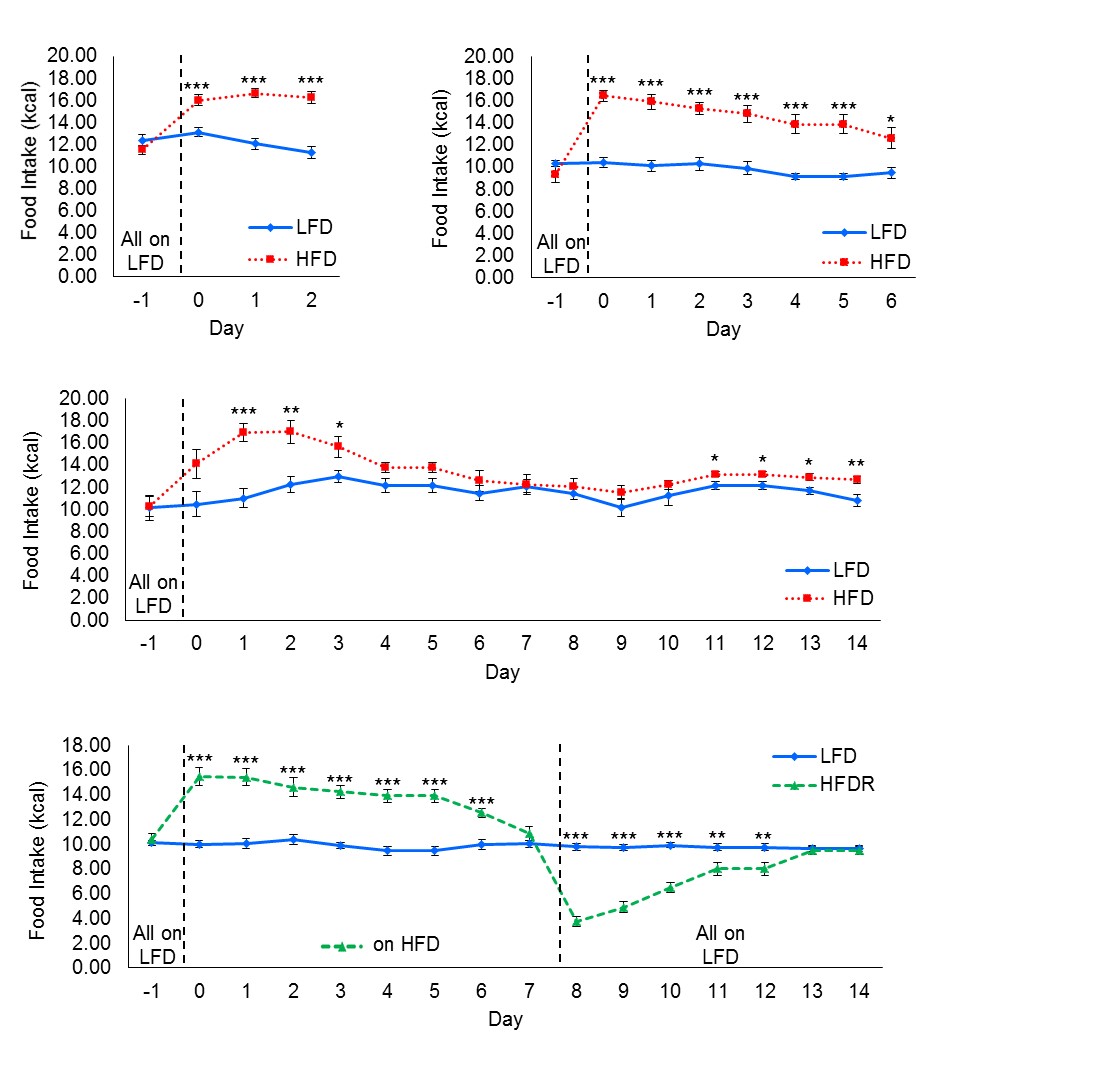

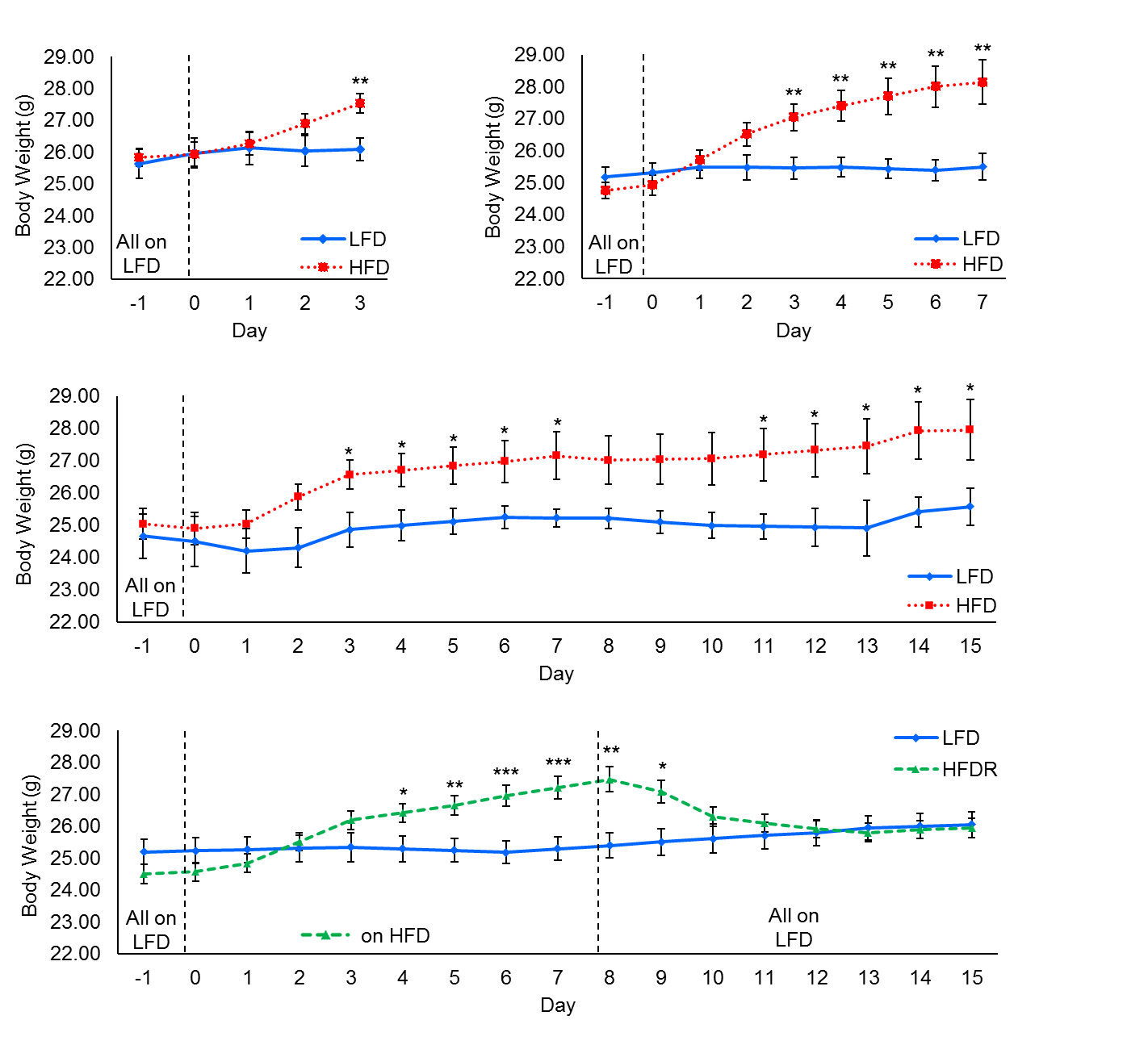


A)

C)

B)

D)

E)

G)

H)

F)

**Figure S2**- Body weights (g) of mice that underwent intraperitoneal glucose tolerance tests (IPGTT). General linear model for repeated measures showed at: A) 3 days of HFD there was no effect of diet (F_(1,14)_=0.901, p=0.359), an effect of time (F_(2.385,33.386)_=27.852, p<0.001) and an interaction (F_(2.385,33.386)_=14.586, p<0.001); B) 1 week of HFD there was an effect of diet (F_(1,14)_=5.600, p=0.033), time (F_(1.605,22.467)_=32.023, p<0.001) and an interaction (F_(1.605,22.467)_=25.862, p<0.001); C) 2 weeks of HFD there was an effect of diet (F_(1,14)_=5.239, p=0.038), time (F_(2.668,37.346)_=11.701, p<0.001) and an interaction (F_(2.668,37.346)_=3.143, p=0.042) and D) 1 week following diet switch (HFDR), there was no effect of diet (F_(1,14)_=1.567, p=0.231), an effect of time (F_(2.620,36.673)_=20.186, p<0.001) and an interaction (F_(2.620,36.673)_=19.569, p<0.001).

Food intake (kcal) of mice that underwent intraperitoneal glucose tolerance tests (IPGTT). General linear model for repeated measures showed at: E) 3 days of HFD there was an effect of diet (F_(1,14)_=96.508, p<0.001), time (F_(3,42)_=11.086, p<0.001) and an interaction (F_(3,42)_=14.133, p<0.001); F) 1 week of HFD there was an effect of diet (F_(1,14)_=37.847, p<0.001), time (F_(3.168,44.359)_=16.113, p<0.001) and an interaction (F_(3.168,44.359)_=14.611, p<0.001); G) 2 weeks of HFD there was an effect of diet (F_(1,14)_=16.240, p=0.001), time (F_(4.552,63.732)_=6.149, p<0.001) and an interaction (F_(4.552,63.732)_=2.965, p=0.021) and H) 1 week following diet switch (HFDR), there was an effect of diet (F_(1,14)_=12.639, p=0.003), time (F_(3.516,49.218)_=46.000, p<0.001) and an interaction (F_(3.516,49.218)_=43.012, p<0.001).

Data is mean ± SEM. All groups n=8. * p<0.05, ** p<0.01, *** p<0.001. Vertical dashed lines indicate the day of diet change but are offset to avoid overlying data points. Intraperitoneal glucose tolerance test (IPGTT), low-fat diet (LFD), high-fat diet (HFD) and high-fat diet recovery (HFDR).
